# Learning from Failure: An Attempt to Measure Dissolution Kinetics of High-Chromia Refractories by Petcoke-Derived Gasification Slags Using In Situ X‑ray Diffraction

**DOI:** 10.1021/acsomega.5c11708

**Published:** 2026-04-10

**Authors:** Shubhadeep Banik, Nichole M. Wonderling, Sarma V. Pisupati

**Affiliations:** † John and Willie Leone Family Department of Energy and Mineral Engineering and The EMS Energy Institute,University Park, Pennsylvania 16802, United States; ‡ Center for Critical Minerals, 8082 The Pennsylvania State University, 407 Academic Activities Building, University Park, Pennsylvania 16802, United States; § Materials Research Institute, The Pennsylvania State University, N-151 Millennium Science Complex, University Park, Pennsylvania 16802, United States

## Abstract

Gasifying petroleum coke (petcoke) offers a promising route to low-carbon energy, hydrogen, and chemical intermediates. However, vanadium- and iron-rich slags aggressively corrode high-chromia refractories in entrained-flow gasifiers, shortening service life and increasing maintenance costs. To address this, we developed an in situ high-temperature X-ray diffraction (HT-XRD) protocol, which involved failure analysis of previous experiments, to attempt the measurement of dissolution kinetics at the liquid petcoke slag/solid high chromia refractory interface under gasifier-relevant conditions (1500 °C, 70% CO–30% CO_2_). Thermodynamic modeling (FactSage 8.3, 1100–1500 °C) predicted Pt stability under the defined conditions, and testing in a furnace on unstrained Pt under similar conditions confirmed these predictions; however, experiments performed on the Pt heating strip on an Anton Paar HTK2000 nonambient chamber contradicted these predictions with premature failure of the Pt heating strip. We believe this is related to interfacial reactions with slag/refractory species occurring under tensile stress. SEM-EDS confirmed Pt interdiffusion with Cr, Al, and Si. The addition of a MgO (110) single crystal between the Pt strip and the refractory eliminated this issue and provided a simple, low-cost solution that enabled stable isothermal runs up to just under 2 h. In theory, XRD peak-area decay of Cr_2_O_3_ should yield time-resolved consumption rates and enable accelerated screening of refractory and slag chemistries. In reality, we were unable to see a significant Cr_2_O_3_ XRD peak decay under the conditions achievable with the available instrumentation. Improvements in nonambient stage design—such as enhanced thermal stability and longer high-temperature operation available on newer versions of this nonambient chamber from Anton Paar and lessons learned from this research could further expand the technique’s utility across industrial corrosion studies.

## Graphical abstract

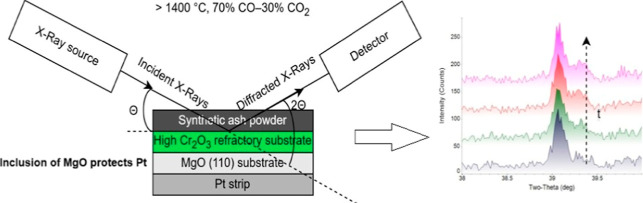

## 1 Introduction

Gasification is a process in which a solid carbonaceous fuel is converted to synthesis gas (or syngas), which has a useable heating value.^1^ Syngas, a mixture of CO and H_2_, is used to generate electricity and chemical products such as CH_3_OH, NH_3_, liquid fuels, and H_2_. In gasification, CO_2_ can also be captured, sequestered, or reused in other applications. Steam can be combined with CO from syngas to form more H_2_ and CO_2_. Therefore, gasification plants that can produce H_2_ and capture and sequester CO_2_ have a lower carbon footprint than conventional methods of burning carbonaceous fuels. Petroleum coke, or petcoke, is a byproduct of petroleum refining. The global production of petcoke has reached 150 million metric tons per annum, and it is anticipated that production will increase in the future.^2^ By gasification, several useful products can be obtained from petcoke. Its heating value is 20% higher than coal, and it has only 0.1–0.3 wt % ash content, whereas coal has 8–20 wt % ash content.^3^ High temperatures (above 1300 °C) in entrained flow gasifiers are necessary for the conversion of petcoke, as its reactivity is lower than that of coal.^4^ The inorganic material after gasification of the feedstock is called ash. It melts above 1400 °C to form slag,^5^ which corrodes the refractory wall in gasifiers. Bennett et al.^6^ reported that, based on nine petcoke slags, the average contents of V_2_O_5_ and Fe_2_O_3_ could be up to 57 and 7.2 wt %, respectively. These oxides are believed to be corrosive to commonly used high chrome oxide refractories.^6^ Although the expected life of the refractory wall is 3 years, the service life can be reduced to as little as three months in some regions of the gasifier, such as the dome, sidewall, and lower cone. Replacement of the refractory lining can cost more than 1 million dollar, depending on the gasifier’s dimensions. At least 10 days of production time are also lost.^6^ Bilbao et al.^7^ explained that the corrosion of porous refractory by liquid slag includes impregnation of slag and chemical reaction between slag and refractory. To simulate corrosion and predict the wear rate of high-chrome oxide refractories, the kinetics of dissolution of the refractories by corrosive V-rich slags under gasification conditions are required.

Several researchers have studied the mechanism of corrosion of high-chrome oxide refractories by coal slags under gasification conditions in recent years.^8–20^ Cr_2_O_3_–Al_2_O_3_–ZrO_2_ refractory bricks, in which the major component was Cr_2_O_3_, were used in most of these studies; Cr_2_O_3_–Al_2_O_3_–ZrO_2_–P_2_O_5_ ^13^ and Cr_2_O_3_–Al_2_O_3_–MgO^20^ refractories were used in two studies. Mg–Fe–Al–Cr,^8,10,11,13,14,18^ Mg–Fe–Cr,^15,17^ and Fe–Al/Cr^12^ spinels formed on the refractory surfaces. According to Gao et al.,^8^ spalling of high-chromia refractory occurred due to slag corrosion and thermal stress. A composite spinel phase [(Mg, Fe^2+^)­(Al, Cr)_2_O_4_], with a high thermal expansion coefficient, was formed in the slag–refractory interface, which contributed to thermomechanical stress. In Na-rich slags, Na decreased the slag surface tension and improved the wettability of the slag on the refractory.^10,16^ Liu et al.^10^ observed that the Na-rich slag penetrated significantly into the refractory; however, there was poor interaction of the slag with the refractory. The main corrosive elements were Fe and Mg. Gao et al.^12^ also studied the interaction of a Na-rich slag with high chromia refractory. Na vapor diffused into the refractory material and caused the chromium oxide aggregate to dissociate. Na-rich slag reacted with refractory material to form low melting point phases (Na_2_CrO_4_, NaAlSiO_4_, NaAlSi_3_O_8_) that reduce the resistance of the refractory to penetration and corrosion. Similar to the work of Liu et al.,^10^ other researchers also observed the corrosion of high-chromia refractory due to Mg and Fe^11,14,15^ by the formation of spinel. Spinel can cause thermomechanical stress in the refractory and cause a reduction in slag penetration. In the study by Zhou. et al.,^14^ spinel formation in experiments performed in air and reducing conditions caused a reduction in slag penetration. Under reducing conditions, corrosion was found to be more severe due to the formation of Cr from Cr_2_O_3_ and its diffusion into the slag. In the study of Chen et al.,^15^ the Cr_2_O_3_–Al_2_O_3_ shell in high chromia refractory reacted with Fe_2_O_3_ from slag and formed a (Cr, Al, Fe)_2_O_3_ solid solution. Further reaction between MgO­(FeO) and (Cr, Al, Fe)_2_O_3_ caused precipitation of (Mg, Fe)­(Fe, Cr)_2_O_3_ spinel adjacent to the Cr_2_O_3_ core. Liu et al.^13^ and Sun et al.^19^ studied the effect of phosphate additives present in high-chromia refractory during corrosion experiments. In the work of Liu et al., the phosphate additives decomposed into gases, such as O_2_ and P_2_O_3_, which diffused into the interior regions near slag–refractory interface and increased oxygen partial pressure. FeO in the slag was oxidized to Fe_2_O_3_, which increased slag viscosity and inhibited slag penetration. Sun et al. observed that aluminous phosphate partially decomposed above 1200 °C and released P_2_O_5_ and phosphate glass. Phosphate migrated under a suitable temperature gradient in the refractory brick and caused densification of the microstructure, which reduced slag infiltration and spalling. In the corrosion studies of high chromia refractory, several authors performed post-mortem analysis on the refractory bricks obtained from slagging gasifiers.^8,10,13,15,17–19^ Peng et al.^9^ and Cai et al.^11^ used the rotary drum furnace test in which slag flows along the refractory lining of the rotating furnace. In some of the studies, researchers have used static tests in which slag is melted in a crucible^12,20^ or on a refractory.^14,16^ To identify the elements and the phases in the slag–refractory interface, the researchers performed post-mortem characterization by scanning electron microscopy–energy dispersive spectroscopy (SEM–EDS), X-ray Diffraction (XRD), and X-ray fluorescence (XRF). Chen et al.^15^ used X-ray computed tomography, which is an imaging technique, to investigate the corroded region of the refractory. Gao et al.^12^ used Laser-induced breakdown spectroscopy (LIBS) to study the slag–refractory interface, in which sodium (Na) was present. The authors state that SEM–EDS and XRF were not fully sensitive to Na. Other studies investigated the corrosion resistance of high-chrome oxide refractory brick exposed to various gasification slags^21^ and additives.^22^ Wang et al.^23^ compared the corrosion resistance of high chromia brick with other refractories by exposing them to a coal gasification slag. Lin et al.^24^ derived theoretical models of corrosion to predict the corrosion rate of the gasifier’s refractory. Soll-Morris et al.^25^ measured the rate of dissolution of spherical alumina particles in Al_2_O_3_–CaO–FeO_*x*_–SiO_2_ gasifier slags by using a confocal scanning laser microscope (CSLM). CSLM is an in situ technique in which the dissolution of oxide particles in slags is recorded using a microscope. CSLM consists of a high-temperature furnace and a confocal microscope. Slag is contained in a Pt crucible. Oxide particles are placed on the slag in their solid form before the experiment, or on the molten slag during the experiment.

Several researchers have reviewed the high-temperature experiments available to determine the extent of corrosion of refractories by molten slags in various areas other than gasification, such as the iron and steel industry,^26–29^ copper production,^30^ glass tanks and cement kilns,^27^ and other applications.^31–33^ Some of the experiments in the early reviews, such as the rotary furnace test, slag impingement test, and induction furnace test, were conducted ex-situ. Here, slag and refractory were either stationary or only one of them would be in motion. In ex-situ tests, post-mortem analysis of the affected area (or depth) of the refractory is performed to determine the corrosion kinetics. In another technique known as the cone fusion method, the melting point of a mixture of ground refractory and slag is measured, which indicates the corrosion resistance of the refractory. The extent of corrosion in a glass tank was determined by recording the temperature variation from thermocouples mounted at various locations on the wall. More recently, Huo et al.^29^ categorized the slag-refractory experiments as visualization and nonvisualization experiments. The nonvisualization experiments were ex-situ in nature, and they comprised the crucible test and finger test. In the crucible test (also called the cup test), the crucible is made of refractory material, and slag is contained in the crucible. In the finger test, refractory samples are partially immersed (“static test”) and stirred in molten slag (“dynamic test”), which is contained in a crucible. The in situ visualization experiments comprised CSLM, single hot thermocouple technology (SHTT), and a Monochromatic light high-temperature visualization system (MLHTVS). In SHTT, slag powder is adhered to a Pt–Rh thermocouple with alcohol, and an oxide particle is placed on the slag when it is molten. The dissolution of the oxide particle is recorded and analyzed. In MLHTVS, monochromatic light is absorbed differently by the slag and the oxide, and this difference is used to characterize the corrosion process. The apparatus consists of a high-temperature furnace with a visual window, a monochromatic light source, and a monochromatic camera. The crucible, made of oxide, must be cut two-thirds along its axial direction before beginning the experiment. This allows the camera to record the corrosion process through the visual window. Xie et al.^34^ developed an in situ gravimetric technique to measure the corrosion kinetics of magnesia spinel refractory in SiO_2_–CaO–Al_2_O_3_–MgO slags. In this method, a crucible containing a slag was brought into contact with a refractory sample suspended from a balance. The gravimetric reading of the balance from the point of initial contact to the end of the experiment indicated the slag’s penetration and dissolution of the refractory. Bilbao et al.^35^ used the high-temperature X-ray diffraction (XRD) technique to study the in situ kinetics of corrosion of high-alumina refractory by Al_2_O_3_–CaO–SiO_2_ slags. Unlike the methods above, the phases that precipitate during reactions between slag and refractory can be monitored in real time using XRD. Powdered refractory was mixed with slag powder before the experiments. So, the experiments did not reproduce the liquid slag–solid refractory interface. This limitation can be overcome, as demonstrated in the study by Dehaven,^36^ in which the reaction kinetics were measured at the liquid Sn/Pb solder–solid metal (Cu and Ni) interface using a high-temperature XRD.

Based on the observations made in the techniques used to measure the kinetics in slag–refractory corrosion in gasification and other fields, in situ high-temperature XRD appears to be the most appropriate and hence was used in this study to perform the experiments between the slag and the porous refractory substrate. The research objective was to measure the in situ kinetics of dissolution of a high-chromium oxide refractory substrate by petcoke slag under conditions typical of an entrained-flow gasifier. Through failure analysis of previous experiments, a protocol was established that allowed to attempt the measurement of the dissolution kinetics at the liquid–solid interface.

## 2 Methodology

### 2.1 Materials

Chemical composition of a petcoke ash was determined using ICP-AES (Inductively coupled plasma atomic emission spectroscopy) (see Table 1). Since petcoke’s mineral content is low,^3^ synthetically prepared ash was used in the experiments. Only the major oxides, which constitute about 94.9 wt % of all oxides, as shown in Table 1, were used to prepare the ash. The inclusion of minor components would cause several minor peaks to appear in the diffraction patterns, making the identification of phases very difficult.

**Table 1:** Composition of Synthetic Petcoke Ash Consisting of All Oxides and Only Major Oxides

component (all oxides)	(wt %)	component (major oxides)	(wt %)
Al_2_O_3_	10.00	Al_2_O_3_	10.54
BaO	0.09	CaO	4.21
CaO	4.02	Fe_2_O_3_	9.17
Fe_2_O_3_	8.70	NiO	14.44
K_2_O	1.85	SiO_2_	10.75
MgO	0.33	V_2_O_5_	50.90
MnO	0.08		
Na_2_O	1.63		
NiO	13.70		
P_2_O_5_	0.65		
SiO_2_	10.22		
SrO	0.04		
TiO_2_	0.44		
V_2_O_5_	48.27		
total	100.00	total	100.00

The composition of the high-chromium oxide refractory used in this study is presented in Table 2. The sum of the weight percentages of the components was 99.5 wt %. The remaining 0.5 wt % consisted of components that the manufacturer did not disclose due to proprietary reasons. Porosity of the refractory was 13%. Substrates with dimensions of 10 mm × 10 mm × 0.5 mm were prepared from the refractory.

**Table 2:** Composition of High Chromium Oxide Refractory

component	(wt %)
Cr_2_O_3_	89.6
Al_2_O_3_	6.6
P_2_O_5_	3.3
total	99.5

During two experiments with the synthetic ash powder and the refractory substrate (see Figure 1a) in a diffractometer (see Section 2.2), the Pt strip of the diffractometer completely cracked. Pt tends to combine with refractory oxides, such as Al_2_O_3_, ThO_2_, and ZrO_2_, at high temperature and in an atmosphere with oxidizing potential below a critical level; MgO is resistant to this type of decomposition.^37^ The low oxidizing potential causes the refractory oxides to dissociate, and thereafter, Pt forms alloys with the metals. So, MgO substrates with dimensions of 10 mm × 10 mm × 0.5 mm and a (110) crystal orientation were placed between the Pt strip and the refractory substrate in the subsequent experiments (see Figure 1b).

**Figure 1:**
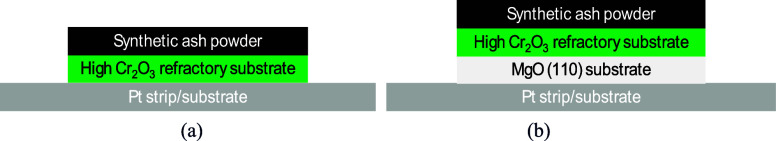
Setup of synthetic ash powder, refractory substrate, MgO substrate, and Pt strip/substrate in (a) original and (b) modified configurations for experiments in the diffractometer and vertical furnace.

### 2.2 XRD

XRD was used to measure the in situ kinetics of dissolution of Cr_2_O_3_ by petcoke slag. A Malvern Panalytical Empyrean diffractometer was used to perform experiments at high temperatures under gasification conditions and to obtain the diffraction pattern in situ. The diffractometer was equipped with a Co anode, line focus optics, X’celerator detector, and Anton Paar HTK2000N nonambient chamber. The nonambient chamber consists of a Pt strip on which the sample is placed. The Pt strip was calibrated by thermal expansion of CeO_2_ up to 1500 °C in air. A synthetic ash powder-ethanol slurry was prepared and spread on the high chromium oxide substrate. After the ethanol evaporated, the substrate was placed on the Pt strip in the diffractometer (see Figure 1a for the original configuration). Following the failed experiment (where the Pt strip cracked), subsequent experiments were performed using the modified configuration (see Figure 1b). The experiments were performed in a 70% CO–30% CO_2_ atmosphere. The volume of the nonambient chamber and the gas flow rate (0.4 SCFH) were used to calculate the time needed to fill the chamber. The gas was allowed to flow for a duration 5 times longer than the estimated time to ensure that air was fully displaced, and the chamber, as well as the inlet and outlet tubing, were filled with the reducing gas. The gas flow rate was maintained for the rest of the experiment. The sample was scanned to align the Pt strip vertically. The Cr_2_O_3_ peak shown in the scan was aligned with the most intense (100%) peak of Cr_2_O_3_ (International Centre for Diffraction Data PDF-5 Powder Diffraction File# 04-005-9887), which appears at 39.513°, in the database.^38^ The sample was heated to 1500 °C at a rate of 60 °C/min and maintained at that temperature for 117 min before the cooling phase. The HTK2000 nonambient chamber can be kept at temperatures above 1400 °C for a maximum duration of 2 h. During the isothermal step, the sample was scanned several times with a step size of 0.0167° over a 2θ range of 37.00–40.98° (Accelerating voltage: 40 kV; current: 40 mA). The time per step was 105 s, and the total scan time for one scan was approximately 5 min.

In chemical reactions on sufficiently thin films, the volume fraction of material consumed at time *t*, *x*(*t*), can be expressed in terms of the X-ray intensities *I*_hkl_ (*h*,*k*,*l* −Miller indices) at time *t* and initial time (*t* = 0) as $x\left(t\right)=-\frac{I_{hkl}\left(t\right)-I_{hkl}\left(0\right)}{I_{hkl}\left(0\right)}$ .^36^ The amount of refractory material dissolved by the slag can be correlated to the area of the peaks of Cr_2_O_3_, which evolve as a function of time. In this study Cr_2_O_3_ peak from the refractory substrate was identified in each scan. The area of a peak in a diffraction pattern refers to the integrated intensity of that peak. Here, *x*(*t*) was calculated by using the area of the Cr_2_O_3_ peaks. Calculation of the area of the peaks of Cr_2_O_3_ and identification of phases in the slag and substrates were done in the software Jade.^39^ The FCJ model was selected in the Profile Shape Functions, and profiles were fitted to the diffraction patterns to calculate the areas of the peaks. The areas were calculated three times for each peak, and their average was used in the analysis.

### 2.3 FactSage

To identify the cause of damage to the Pt strip, thermodynamic simulations were performed in the Equilib module of FactSage 8.3.^40^ FToxid and FactPS databases were selected. The objective of these simulations was to determine the phases formed in equilibrium calculations between the refractory and Pt substrates in air (79% N_2_–21% O_2_) and a reducing atmosphere (70% CO–30% CO_2_) at temperatures ranging from 1100 to 1500 °C. In another set of simulations, MgO was introduced in the original combination of refractory and Pt substrates to determine whether Pt and/or refractory substrates form any phases with MgO.

### 2.4 Vertical Furnace

A vertical furnace with natural draft was used to perform two experiments with the synthetic ash powder, refractory substrate, MgO substrate, and Pt substrate in the original and modified configurations. These experiments were intended to determine whether the inclusion of MgO substrate in the modified configuration can protect the Pt strip. Pt substrates were obtained from the Pt strip that was damaged in the previous experiment with the original configuration. A synthetic ash powder coated on a high-chromium oxide refractory substrate (as explained in Section 2.2, XRD) was used. In the first and second experiments, the substrates were arranged in the original configuration (see Figure 1a) and the modified configuration (see Figure 1b), respectively. In both experiments, the combination of substrates was placed in an alumina crucible, which was placed inside another alumina crucible. The second alumina crucible was included to contain any spills from the samples and avoid damage to the furnace. The second alumina crucible was placed on the platform for samples inside the furnace. A third alumina crucible was inverted over the former crucibles. The third crucible was included to reduce leakage of the gas outside the furnace. The samples were heated to 1500 °C in 5% H_2_–95% N_2_ atmosphere at a rate of 10 °C/min. In our previous study,^41^ it was explained that 5% H_2_–95% N_2_ gas mixture has a very similar effect on oxide mixtures as 70% CO–30% CO_2_, which was used in the experiments in the diffractometer. The vertical furnace is not equipped to use a 70% CO–30% CO_2_ gas mixture. The samples were maintained at 1500 °C for 2 h and then cooled down to room temperature.

### 2.5 Scanning Electron Microscopy-Energy Dispersive Spectroscopy (SEM-EDS)

The cracked Pt strip, with attached sample, was imaged and analyzed in the Verios G4 SEM (Thermo-Scientific, Hillsboro, OR) with EDS analysis using the X-MaxN detector (Oxford Instruments, Concord, MA) to determine the elements present in the cracked region.

## 3 Results and Discussion

Figure 2a,b) shows the results of the equilibrium calculations in FactSage in 70% CO–30% CO_2_ atmosphere with (a) Pt and refractory substrates and (b) Pt, MgO, and refractory substrates. In these calculations, the initial mass of the substrates was set to 1 g. The mass of the gases was set to 70 g CO and 30 g CO_2_ to maintain the proportion of the gas mixture. Gas flow rates could not be provided in the Equilib module in FactSage. In Figures 2a and 3a, Al_2_O_3_ and Cr_2_O_3_, which were present in the refractory substrate, were the only phases present in the phase M_2_O_3_ (Corundum). The mass of M_2_O_3_ was almost the same at all temperatures. Mass of Pt(s) (“s” refers to solid) remained at 1 g at all temperatures. P_2_O_5_ from the refractory substrate was transformed into the gas phase. The results of the effect of including a MgO substrate are shown in Figure 2b. The results show that the following phases of Mg from the refractory substrate were present in spinel—Mg_*x*_Al_*y*_O_4_, Mg_*x*_Cr_*y*_O_4_. Mg_3_P_2_ *O*_8_(*s*) appeared at 1100—1300 °C, and at higher temperatures, MgO and P_2_O_5_ appeared in the phase Slag-liq. The mass of Pt was 1 g at all temperatures.

**Figure 2:**
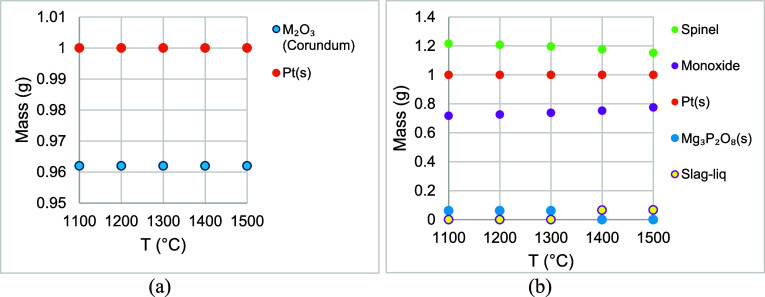
Masses of phases with respect to temperature in equilibrium calculations in 70% CO–30% CO_2_ atmosphere with (a) Pt and refractory substrates and (b) Pt, MgO, and refractory substrates.

**Figure 3:**
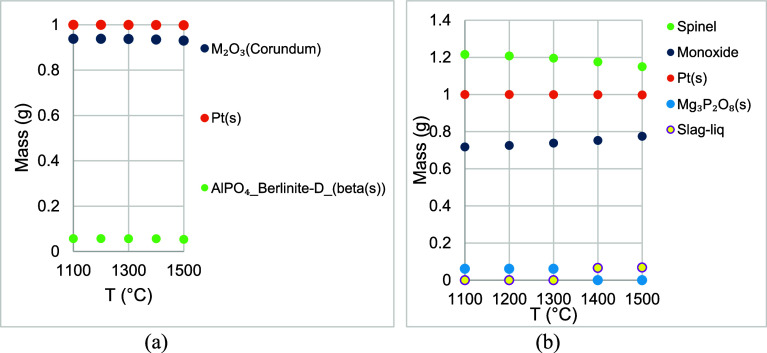
Masses of phases with respect to temperature in equilibrium calculations in 79% N_2_–21% O_2_ atmosphere (air) with (a) Pt and refractory substrates and (b) Pt, MgO, and refractory substrates.

Figure 3a,b shows the results of the equilibrium calculations in FactSage in a 79% N_2_–21% O_2_ atmosphere (air) with (a) Pt and refractory substrates and (b) Pt, MgO, and refractory substrates. In Figure 3a, the masses of M_2_O_3_ (Corundum) and Pt(s) were almost the same at all temperatures. Mass of Pt(s) dropped from 0.9999 g at 1100 °C to 0.99773 g at 1500 °C. Al_2_O_3_ and P_2_O_5_ from the refractory substrate formed the phase AlPO_4__Berlinite-D_(beta(s)). In Figure 3b, the results were similar to those of Figure 2b. Mg_*x*_Al_*y*_O_4_ and Mg_*x*_Cr_*y*_O_4_ were present in spinel. Mg and P were present in Mg_3_P_2_O_8_ between 1100 and 1300 °C, and in Slag-liq between 1400 and 1500 °C. Mass of Pt(s) decreased from 0.9999 g at 1100 °C to 0.99773 g at 1500 °C. Pt*O*_2_(*g*) was present in the simulation results in the gas phase at 1100–1500 °C, and its fugacity was between 10^–7^ and 10^–6^ atm. Its fugacity increased as temperature increased. In the FactSage simulations, it was observed that Pt was affected by the presence of air but stable in a reducing atmosphere. The results from FactSage were not in agreement with the work of Darling et al.^37^ The authors studied the interactions of Pt with Al_2_O_3_, ZrO_2_, and ThO_2_ separately and concluded that Pt was affected by those refractory oxides. In this study, the refractory substrate consisted of a combination of oxides (Cr_2_O_3_, Al_2_O_3_, and P_2_O_5_). In another study by Darling et al.,^42^ it was concluded that geometry also played a crucial role in their experiments. Oxygen must escape from the Pt-refractory interface for the Pt–metal alloys to form. Physical geometry cannot be provided in equilibrium calculations in FactSage. Figures 2b and 3b showed that MgO did not form any phase with Pt, which was an encouraging result for the use of MgO in the diffractometer.

Since results from the FactSage simulations could not confirm whether Pt was reacting with the components of the refractory substrate, experiments and post-mortem analysis were performed to determine the actual cause of the damage to the Pt strip. The Pt substrates, used in experiments with original and modified configurations (see Figure 1) in the vertical furnace, were similar in appearance after the tests. The Pt substrate tested using the original configuration did not crack. The setup inside the furnace did not completely reproduce the setup inside the diffractometer. The Pt strip inside the diffractometer was held under a specified tension, whereas the Pt substrate inside the furnace was placed on the floor of the crucible. The presence of the applied tension could be significant in causing rupture after reaction between the Pt strip and the refractory substrate at high temperature.

**Figure 4:**
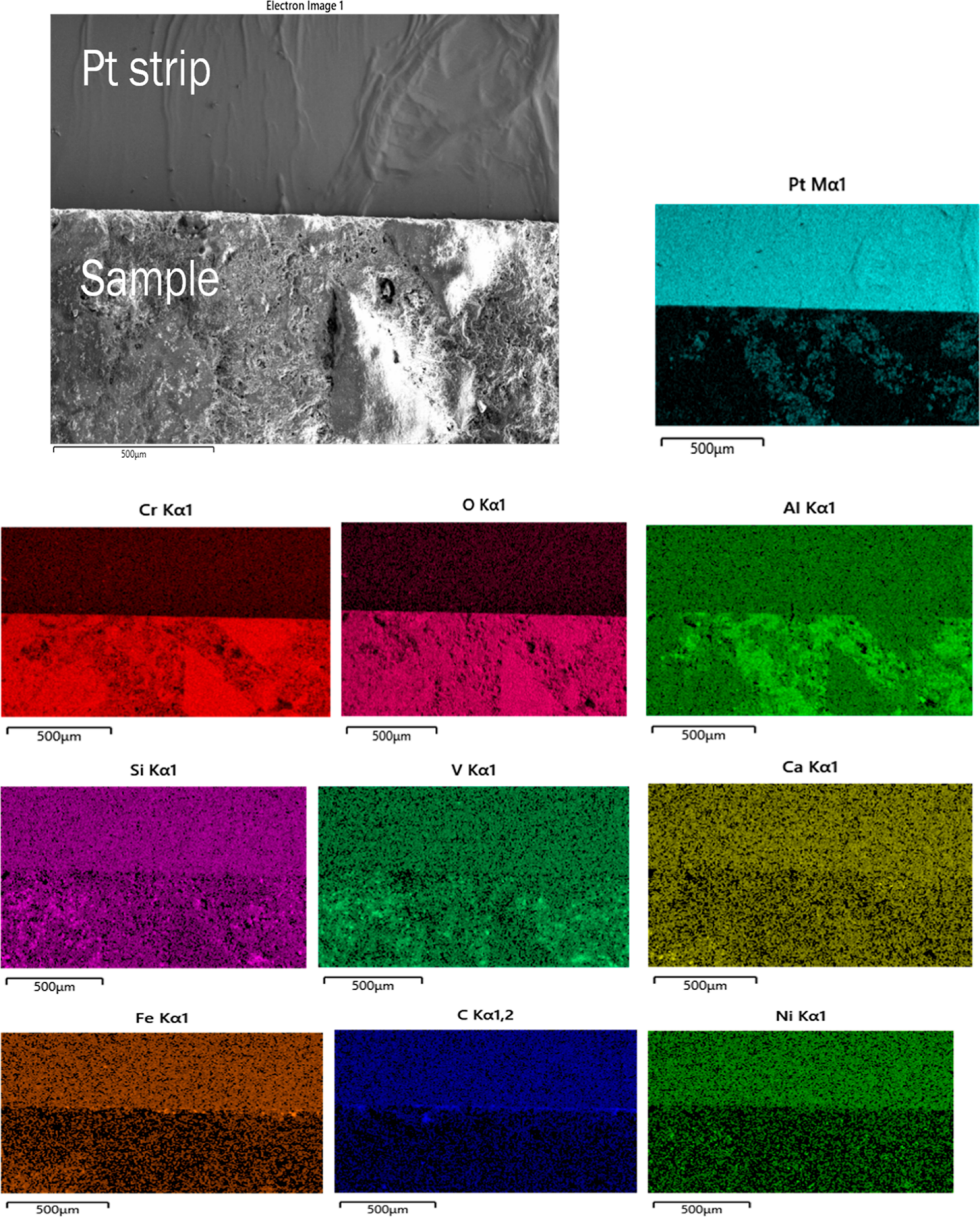
SE image and EDS map scan, depicting elemental distribution, of cracked Pt strip with attached sample.

**Figure 5:**
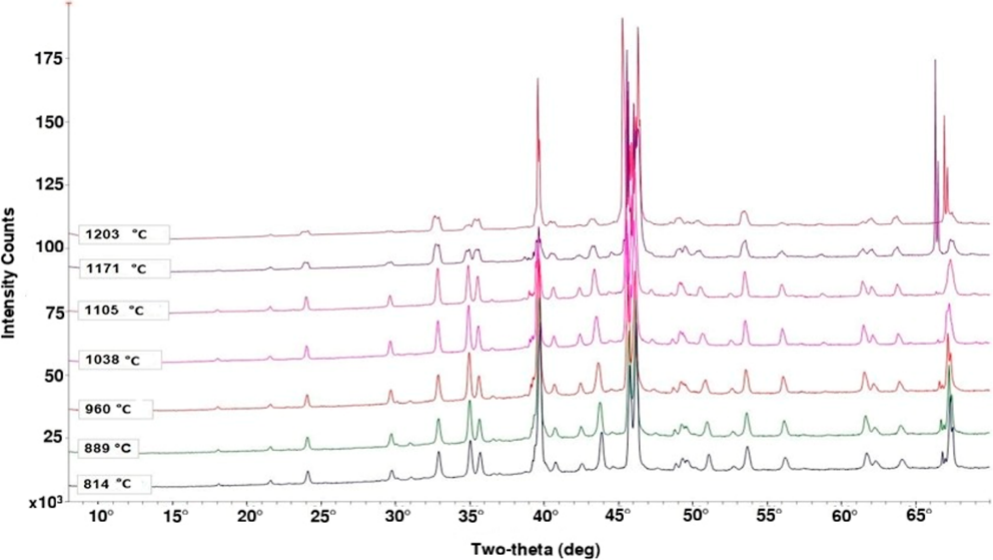
Diffraction patterns of synthetic petcoke ash in 70% CO (balance CO_2_) atmosphere.

**Figure 6:**
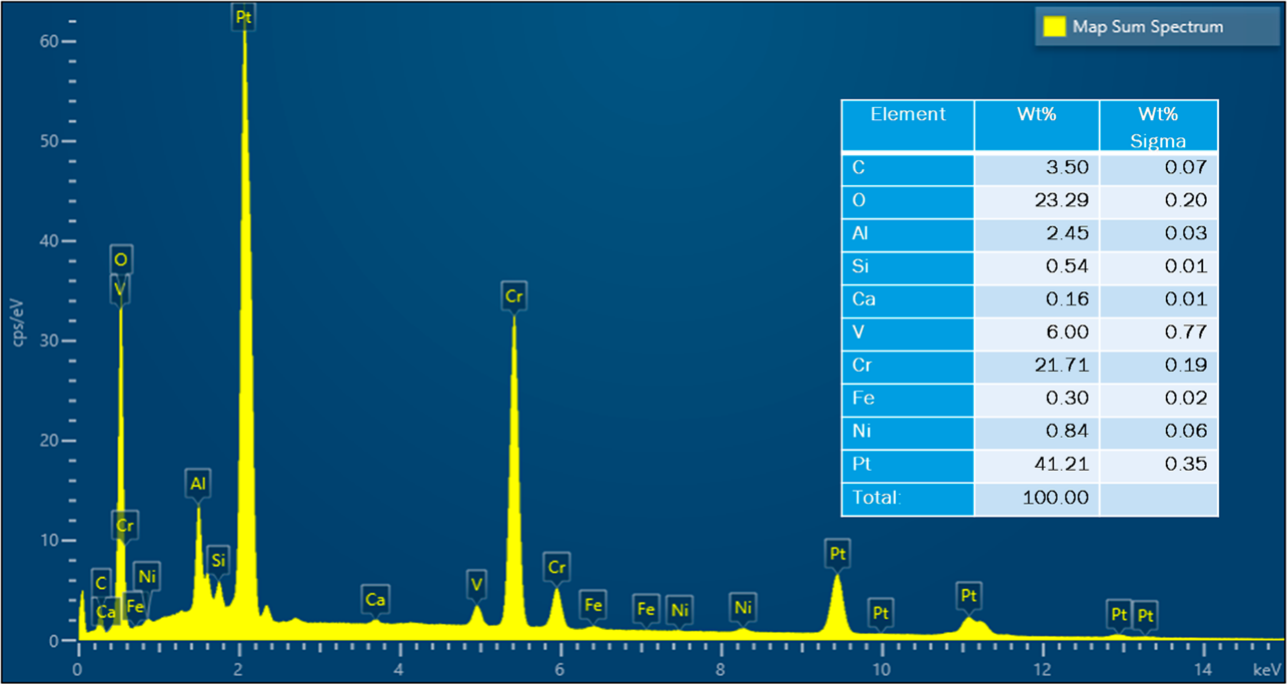
Acquired EDS sum spectrum of map scan on cracked Pt strip with attached sample.

**Figure 7:**
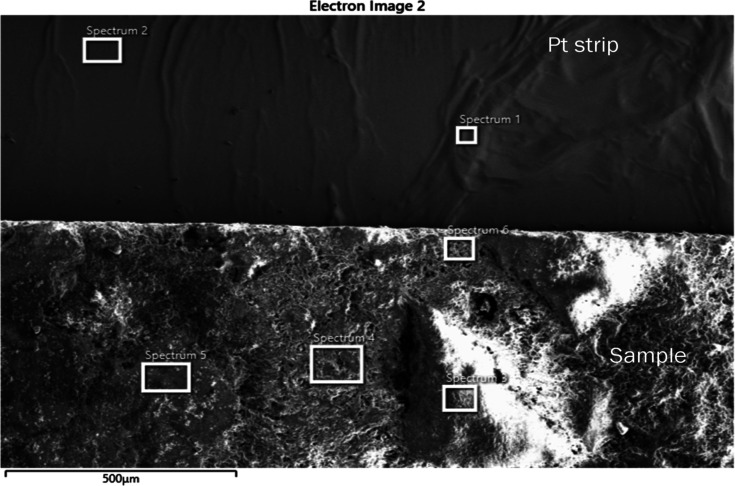
SE image of cracked Pt strip, with attached sample, showing locations where spectra were acquired.

**Figure 8:**
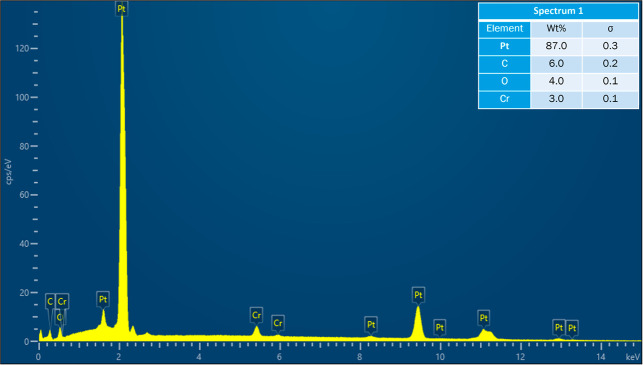
Acquired EDS spectrum at location 1 on cracked Pt strip.

**9:**
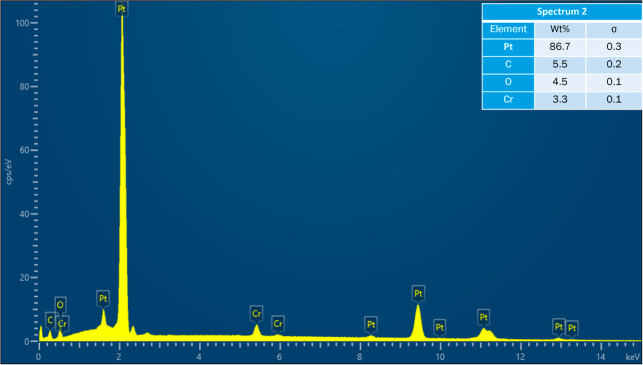
Acquired EDS spectrum at location 2 on cracked Pt strip.

**Figure 10:**
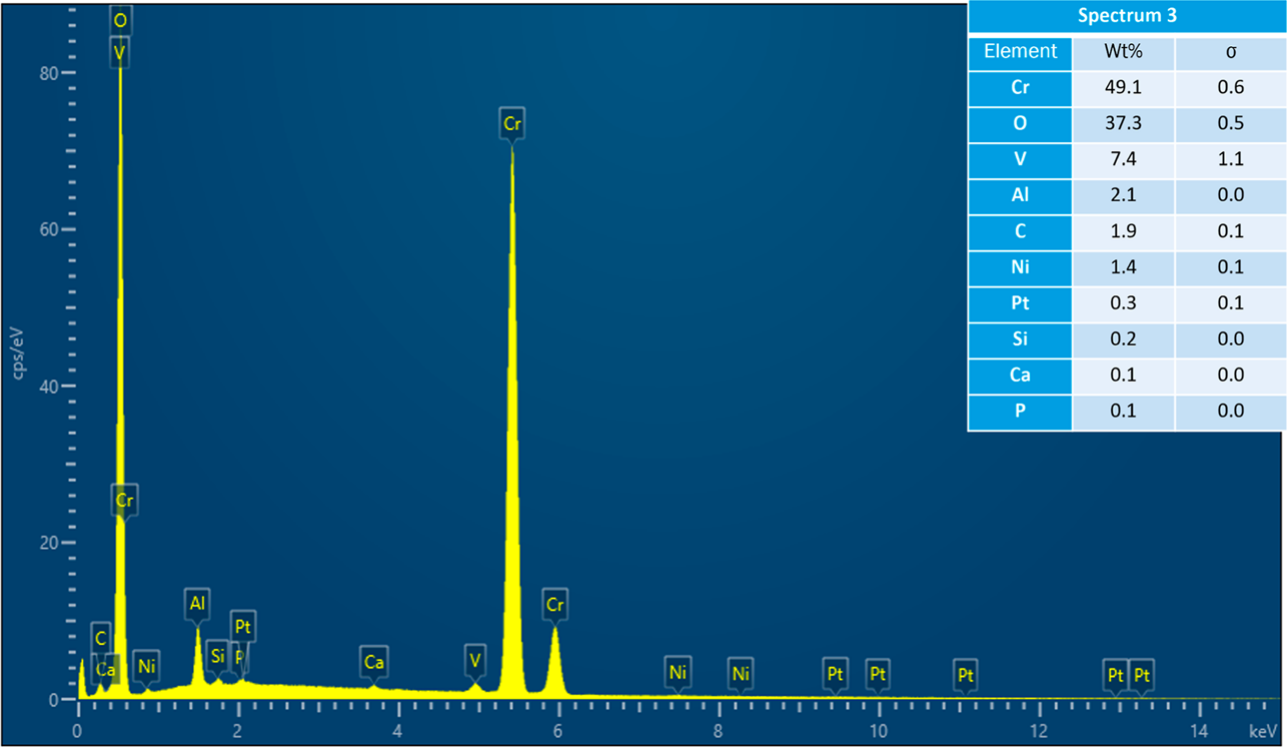
Acquired EDS spectrum at location 3 on reacted sample.

**Figure 11:**
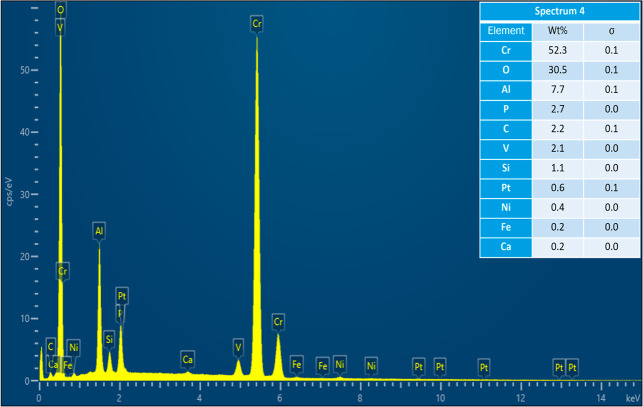
Acquired EDS spectrum at location 4 on reacted sample.

**12:**
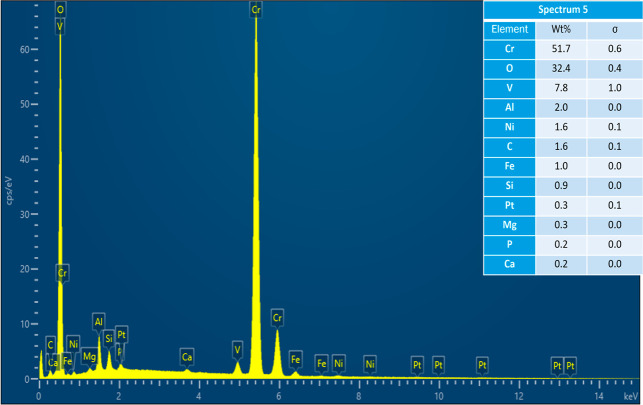
Acquired EDS spectrum at location 5 on reacted sample.

As the results of experiments in the vertical furnace were not conclusive regarding any reaction between the Pt strip and the refractory substrate, damaged Pt strips from experiments in the diffractometer were analyzed in SEM–EDS. Figure 4 shows the secondary electron (SE) image and the EDS map scan of the cracked Pt strip with attached sample. The sample is a piece of the refractory substrate that reacted and adhered to the Pt strip. Pt content was high in the Pt strip region. There was also some Pt in the sample region, which indicates that Pt reacted with the sample. Cr, O, and Al were present in the refractory substrate (see Table 2). So, their content was high in the sample region. Al, Si, V, Ca, Fe, and Ni were present in the petcoke ash composition (see Table 1). These elements were present in both the Pt strip and the sample regions. The refractory substrate was coated with the ash at the beginning of the experiment. Some of the ash or slag possibly migrated toward the Pt strip region during the experiment. Diffraction patterns of the same synthetic petcoke ash were successfully measured up to 1203 °C in the same instrument in our previous study.^43^ Of relevance to the present work, the diffraction patterns from 814 to 1203 °C are shown here in Figure 5, as only diffraction patterns below 814 °C were included in the aforementioned article. The acquired EDS sum spectrum of the map scan is shown in Figure 6. EDS spectra were also acquired at six locations on the Pt strip and the sample region, as shown in Figure 7, to identify the elements responsible for reaction with Pt. The acquired EDS spectra on the six locations are shown in Figures 8–13. The weight percent of all the elements detected in all spectra is shown in Table 3. Cr was present in Spectra 1 and 2, which were acquired in the Pt strip region. So, Cr reacted with the Pt strip. Locations 3, 4, and 5 were less than 500 μm away from the Pt-sample interface, and location 6 was almost at the interface. In all the locations, Pt was detected, along with Cr, Al, and Si. These elements were present in the refractory substrate and the petcoke ash. Cr can react with Pt, as seen in spectra 1 and 2 in the Pt strip. Darling et al.^37^ stated that alumina refractory with siliceous impurities can embrittle Pt wires at high temperatures (above 1200 °C) in reducing atmospheres. Among the locations 3–6, Pt was highest in location 6 as it was closest to the interface.

**Table 3:** Weight Percent of Elements Detected in All Spectra

spectrum label	1	2	3	4	5	6
element	Wt %					
C	6.00	5.53	1.91	2.22	1.56	7.70
O	3.98	4.50	37.29	30.47	32.40	30.20
Mg					0.26	0.12
Al			2.13	7.69	1.99	8.85
Si			0.19	1.14	0.91	1.26
P			0.10	2.69	0.23	5.07
Ca			0.15	0.16	0.18	0.23
V			7.44	2.11	7.77	3.73
Cr	2.97	3.26	49.13	52.26	51.73	38.44
Fe				0.23	0.99	1.74
Ni			1.38	0.45	1.64	1.57
Pt	87.05	86.70	0.27	0.60	0.33	1.08
total	100.00	100.00	100.00	100.00	100.00	100.00

**Figure 13:**
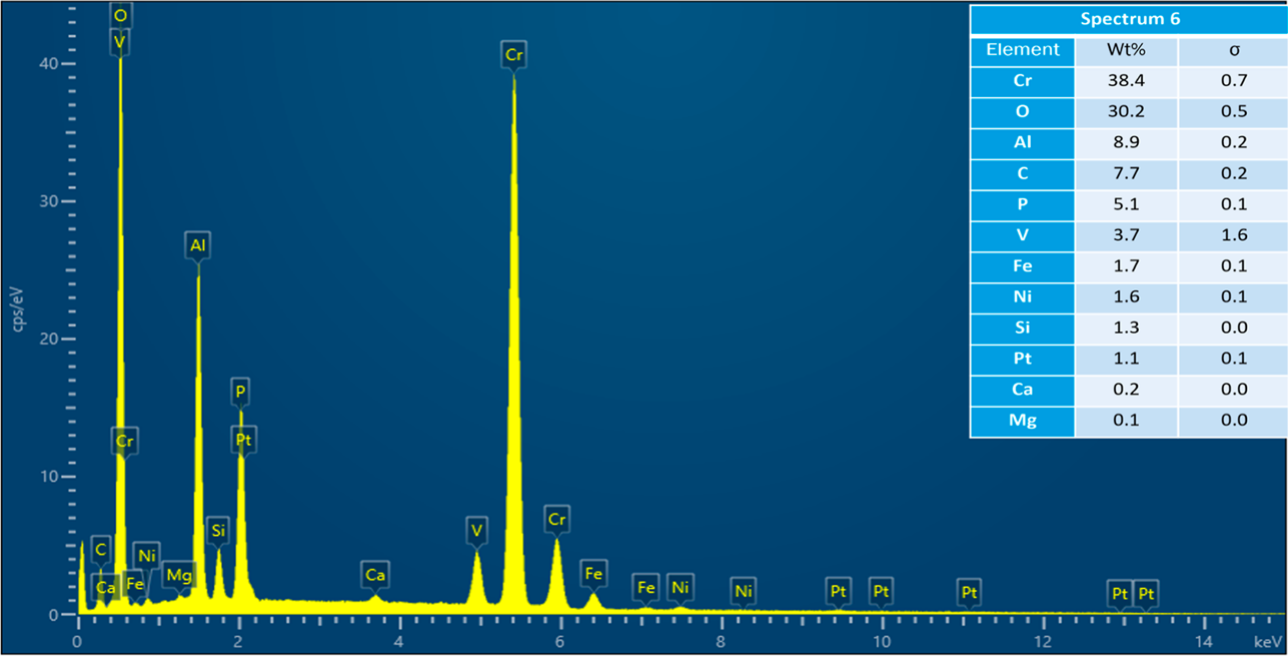
Acquired EDS spectrum at location 6 on reacted sample.

Measurement of diffraction patterns on the synthetic petcoke ash at 1500 °C in the original configuration (see Figure 1a) was attempted twice, and the Pt strip cracked on both occasions. (see Figures 10 and 11) Based on the results of FactSage simulations, experiments in the vertical furnace, and SEM–EDX spectra, it was decided to continue the remaining experiments in the diffractometer at high temperatures in the modified configuration (see Figure 1b). Several experiments were performed at high temperatures. With the inclusion of the MgO substrate, it was possible to measure the diffraction patterns on the synthetic petcoke ash at high temperatures. Figure 14a,b) shows the overlay of diffraction patterns measured from synthetic petcoke ash–high chromia refractory at 1500 °C in 70% CO–30% CO_2_ atmosphere in two experiments. These are denoted as “Experiment 1” and “Experiment 2”. In Experiment 2, the sample was scanned five times. The diffraction patterns were stacked in order of increasing reaction time. The Cr_2_O_3_ peak, identified at 39.513° at room temperature, shifted at high temperatures due to deformation of the Pt strip at those temperatures. Figure 15 shows the overlay of measured diffraction patterns at 1400 °C of synthetic petcoke ash. The diffraction patterns were stacked in order of increasing reaction times. In another experiment, in which the sample was maintained at 1400 °C under a 70% CO–30% CO_2_ atmosphere, the sample was scanned after the cooling stage at ambient temperature under a reducing atmosphere. The diffraction pattern from that scan, shown in Figure 16, illustrates the effect of the reducing gases on the ash/slag. Fe_2_O_3_ was initially present in the synthetic petcoke ash (see Table 1). In Figure 16, the presence of Fe_3_Al indicates that Fe_2_O_3_ was reduced to metallic Fe before it formed a phase with Al. The effect of reducing gas on the ash or slag was also confirmed in our previous study.^43^ Here, the same synthetic petcoke ash was heated in an alumina crucible to 1500 °C under a 70% CO–30% CO_2_ atmosphere in our in-house high-temperature viscometer. The diffraction pattern of the solidified slag post viscometer test showed that NiO present in the synthetic petcoke ash was reduced, and it formed a phase with Al (Ni_0.92_Al_0.08_).

**Figure 14:**
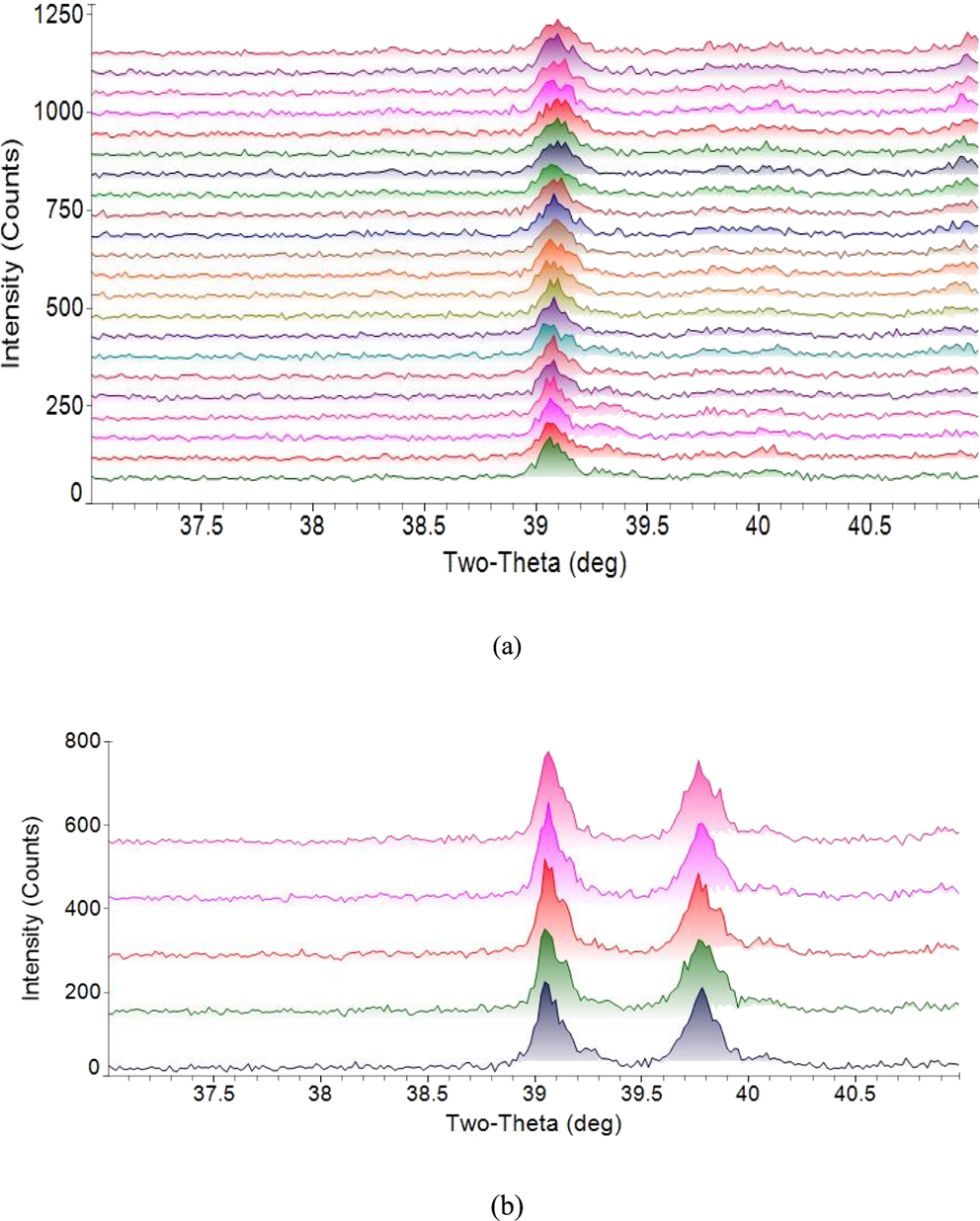
Overlay of measured diffraction patterns at 1500 °C of synthetic petcoke ash placed on high chromium oxide refractory substrate, stacked in order of increasing reaction time—(a) experiment 1, (b) experiment 2.

**Figure 15:**
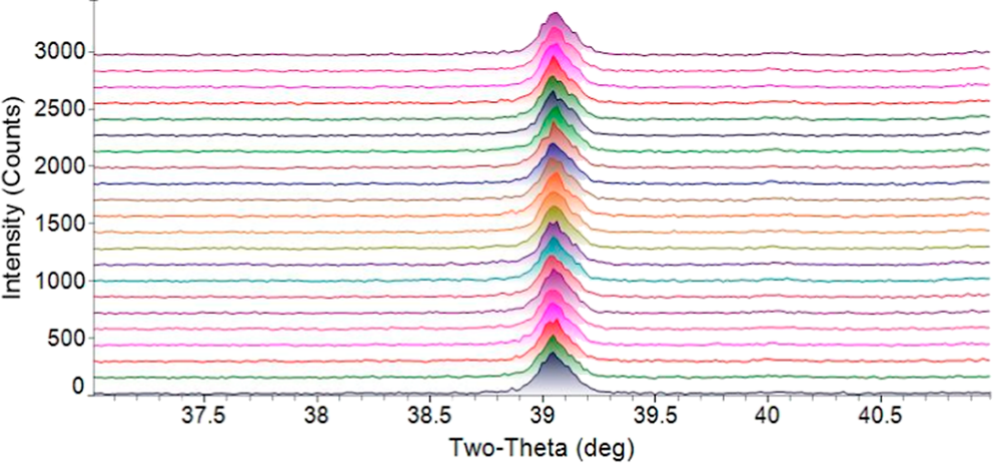
Overlay of measured diffraction patterns at 1400 °C of synthetic petcoke ash placed on high chromium oxide refractory substrate, stacked in order of increasing reaction time.

**Figure 16:**
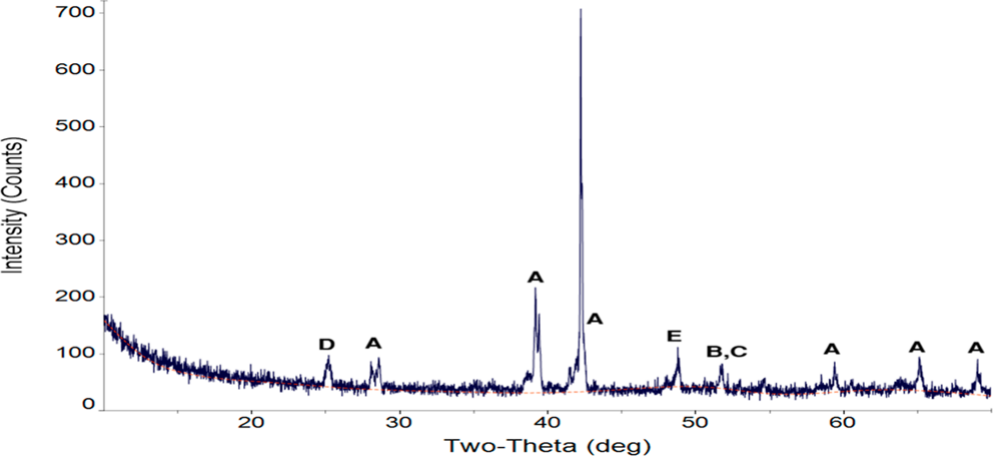
Diffraction pattern of synthetic petcoke ash slag that reacted on high chromium oxide refractory substrate at 1400 °C—diffraction pattern measured postcooling to ambient temperature; A: Cr_2_O_3_, B: Fe_3_Al, C: CaSiO_3_, D: SiO_2_, E: V_0.75_Al_0.25_.

In Figures 14 and 15, the overlays of diffraction patterns could not indicate whether the Cr_2_O_3_ peak at about 39° was decaying as the reaction progressed. In all the overlays, the Cr_2_O_3_ peaks in any diffraction pattern qualitatively appeared very similar to the Cr_2_O_3_ peak in the initial diffraction pattern. This would indicate that the slag did not dissolve Cr_2_O_3_ from the refractory substrate. To confirm this hypothesis, areas of the Cr_2_O_3_ peaks in all overlays were calculated and compared with the area of the Cr_2_O_3_ peak at the beginning of the reaction between the slag and the refractory substrate. The calculated areas are shown with respect to the time of reaction between the slag and the refractory substrate at 1500 °C (experiment 1 and experiment 2) and 1400 °C in Figure 17a–c. If the Cr_2_O_3_ peak had decayed, then the calculated areas would have decreased with respect to time of reaction. However, the areas changed haphazardly with respect to the time of reaction. Normalized relative changes in Cr_2_O_3_ peaks’ areas were calculated according to *x*(*t*) (see Section 2.2), which represents the amount of the refractory substrate dissolved by the slag. Distribution of *x*(*t*) with respect to time of reaction between the slag and the refractory substrate at 1500 °C (experiment 1 and experiment 2) and 1400 °C is shown in Figure 17d–f). According to the equation for *x*(*t*), when a material is being consumed over a period of time, *x*(*t*) should be completely positive and increase during that period. No such trend was observed in Figure 17d–f, which supports the hypothesis that the slag did not dissolve Cr_2_O_3_ from the refractory substrate.

**Figure 17:**
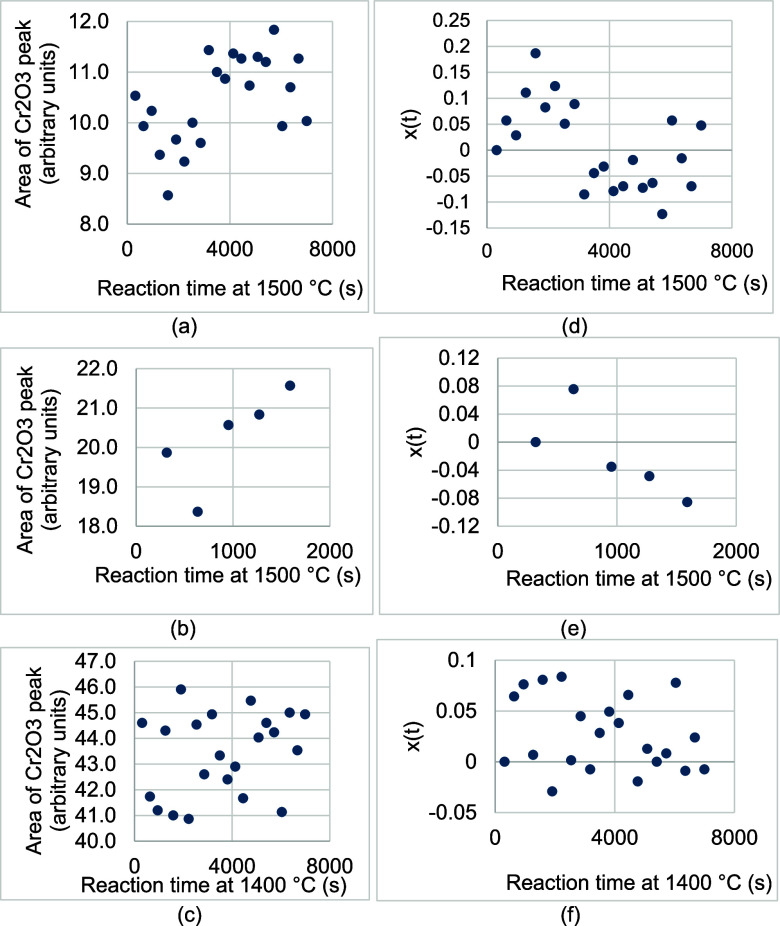
Area of Cr_2_O_3_ peak with respect to time of reaction between synthetic petcoke slag and high-chromium oxide refractory substrate at (a) 1500 °C (experiment 1), (b) 1500 °C (experiment 2), (c) 1400 °C; *x*(*t*) with respect to time of reaction between the slag and the refractory substrate at (d) 1500 °C (experiment 1), (e) 1500 °C (experiment 2), (f) 1400 °C.

Several limitations in the existing diffractometer and in more advanced designs were identified as contributing to the failure in measuring the dissolution kinetics. These limitations included nonhomogeneous temperature distribution, possible interaction of the reaction gas with the heating elements, and limited hold times at high temperatures. A study by Soll-Morris et al.^25^ also provided insights to understand the absence of dissolution in the current study. In their study, dissolution of solid spherical alumina particles (porosity = 0%) was investigated in synthesized gasifier slags at temperatures between 1200 and 1300 °C by using a CSLM. The extent of dissolution was determined by measuring the change in particle radii during the experiments. At 1200 °C, there was no appreciable change in the radius over the entire duration of the experiment. At 1300 °C, the radius dropped slowly, whereas at 1400 °C, the radius dropped rapidly. The authors suggested that at 1200 °C, the slag was saturated with Al_2_O_3,_ which was initially a constituent of the slag. Therefore, there was no dissolution of the alumina particles. Since dissolution of refractory material in a gasifier slag is sensitive to temperature, it is possible that the temperature distribution inside the nonambient chamber of the diffractometer could have affected the interaction between the petcoke slag and the high chromium oxide refractory substrate, although the slag did not consist of Cr_2_O_3_ initially. In the nonambient chamber used for this work, only the Pt strip is heated. Advanced nonambient chambers for diffractometers, such as the Anton Paar HTK 1500, can provide a homogeneous temperature distribution. The HTK 1500 is an environmental heater, which means heating elements heat the chamber. It can reach up to 1500 °C only in air, vacuum, and inert gases.^44^ In other high-temperature instruments, such as our in-house viscometer, the heating elements are not exposed to the gas as the elements heat the tube inside which the gas flows. The nonambient chamber used in this study can be maintained above 1400 °C for a maximum duration of 2 h. Longer hold times could result in appreciable decay of the Cr_2_O_3_ peak. In the study by Soll-Morris et al., at 1300 °C, the particle’s radius initially was almost the same as the original radius, but it decreased slowly as time progressed.

## 4 Conclusions

1. An HT-XRD protocol was established to quantify in situ dissolution of high-chromia refractory by petcoke slag at 1500 °C under 70% CO–30% CO_2_ atmosphere using peak-area decay of Cr_2_O_3_.
2. Early Pt-strip failures originated from interfacial reactions with refractory/slag species under tensile stress; a simple MgO (110) interlayer prevented Pt degradation without adverse chemistry.
3. FactSage predicted phases between Mg and refractory constituents, and Pt stability in reducing atmospheres, but could not capture geometry-dependent embrittlement observed experimentally. EDX spectra acquired at several locations on the cracked Pt strip showed that Cr, Al, and Si reacted with Pt. More importantly, MgO did not form any phase with Pt, which was a very positive result for the subsequent experiments in which a MgO substrate with a (110) crystal orientation was placed between the refractory substrate and the Pt strip.
4. The method can yield phase-specific, time-resolved kinetics at a real slag/refractory interface and enable accelerated screening of refractory and slag chemistries for entrained-flow petcoke gasifiers, provided that improvements in the diffractometer are implemented.
5. Improvements in the design of diffractometers that allow longer duration at high temperatures, and a homogeneous temperature distribution can make this technique even more useful to measure in situ high-temperature corrosion kinetics in various slag–refractory interactions.
